# DNA Methylation Machinery in Gastropod Mollusks

**DOI:** 10.3390/life14040537

**Published:** 2024-04-22

**Authors:** Laura Haidar, Marius Georgescu, George Andrei Drăghici, Ioan Bănățean-Dunea, Dragoș Vasile Nica, Alina-Florina Șerb

**Affiliations:** 1 Department of Functional Sciences, Physiology Discipline, Faculty of Medicine, “Victor Babeș” University of Medicine and Pharmacy Timișoara, Eftimie Murgu Square No. 2, 300041 Timişoara, Romania; haidar.laura@umft.ro; 2 Center of Immuno-Physiology and Biotechnologies (CIFBIOTEH), “Victor Babeș” University of Medicine and Pharmacy Timișoara, Eftimie Murgu Square No. 2, 300041 Timişoara, Romania; 3 Faculty of Pharmacy, “Victor Babeș” University of Medicine and Pharmacy Timișoara, Eftimie Murgu Square No. 2, 300041 Timișoara, Romania; draghici.george-andrei@umft.ro; 4 Research Center for Pharmaco-Toxicological Evaluations, Faculty of Pharmacy, “Victor Babeș” University of Medicine and Pharmacy Timișoara, Eftimie Murgu Square No. 2, 300041 Timișoara, Romania; 5 Biology and Plant Protection Department, Faculty of Agriculture, University of Life Sciences “King Mihai I” from Timișoara, Calea Aradului 119, 300645 Timișoara, Romania; ioan_banatean@usab-tm.ro; 6 The National Institute of Research—Development for Machines and Installations Designed for Agriculture and Food Industry (INMA), Bulevardul Ion Ionescu de la Brad 6, 077190 București, Romania; 7 Department of Biochemistry and Pharmacology, Biochemistry Discipline, “Victor Babeș” University of Medicine and Pharmacy Timișoara, Eftimie Murgu Square No. 2, 300041 Timișoara, Romania; aserb@umft.ro

**Keywords:** DNA methylation, dynamics, gastropods, mollusks, growth, development, reproduction, memory, phenotype

## Abstract

The role of DNA methylation in mollusks is just beginning to be understood. This review synthesizes current knowledge on this potent molecular hallmark of epigenetic control in gastropods—the largest class of mollusks and ubiquitous inhabitants of diverse habitats. Their DNA methylation machinery shows a high degree of conservation in CG maintenance methylation mechanisms, driven mainly by DNMT1 homologues, and the presence of MBD2 and MBD2/3 proteins as DNA methylation readers. The mosaic-like DNA methylation landscape occurs mainly in a CG context and is primarily confined to gene bodies and housekeeping genes. DNA methylation emerges as a critical regulator of reproduction, development, and adaptation, with tissue-specific patterns being observed in gonadal structures. Its dynamics also serve as an important regulatory mechanism underlying learning and memory processes. DNA methylation can be affected by various environmental stimuli, including as pathogens and abiotic stresses, potentially impacting phenotypic variation and population diversity. Overall, the features of DNA methylation in gastropods are complex, being an essential part of their epigenome. However, comprehensive studies integrating developmental stages, tissues, and environmental conditions, functional annotation of methylated regions, and integrated genomic-epigenomic analyses are lacking. Addressing these knowledge gaps will advance our understanding of gastropod biology, ecology, and evolution.

## 1. Introduction

Epigenetics is the branch of genetics that studies changes in gene function which occur without affecting the primary sequence of deoxyribonucleic acid (DNA) [1]. Our current understanding of epigenetic gene regulation is based on mechanistic insights of DNA modifications, histone variants and their modifications, and non-coding ribonucleic acid (RNA) molecules [2,3]. Some epigenetic modifications are relatively stable and can facilitate short-term (mitotic) and long-term (meiotic) transmission of a silent or active gene status. Such changes are often involved in cellular differentiation during development. Thus, once a cell becomes specialized (such as a liver cell or a nerve cell), it maintains its specific epigenetic marks to ensure the appropriate gene expression patterns [3]. Other epigenetic modifications, by contrast, are reversible, meaning they can be altered in response to environmental cues or other signals. The reversible nature of these changes allows cells to adapt to different conditions [4]. One well-known example is DNA methylation, where methyl (-CH_3_) groups can be added or removed from DNA molecules, influencing gene expression [4,5].

Methylation of the genome (methylome) is a potent molecular hallmark of epigenetic control. In particular, methylation at the 5-carbon of the cytosine ring-5-methylcytosine (5mC), also referred to as the “fifth base” of DNA, is the best-characterized DNA modification in animals and is primarily associated with stable, long-term transcriptional silencing [5]. This molecular event is catalyzed by DNA methyltransferases (DNMTs); these enzymes are involved in preserving cytosine methylation during DNA replication (maintenance methylation) and in establishing new DNA methylation patterns (de novo methylation) [6]. The Tet Methylcytosine Dioxygenases (TETs) mediate the oxidation of 5mC to 5-hydroxymethylcytosine (5hmC), and subsequently to 5-formylcytosine (5fC), and then to 5-carboxycytosine (5caC) [7]. The latter two modified nucleotides are then reverted back to 5mC via base excision repair (BER) glycosylases and/or thymine DNA glycosylase (TDG)-mediated base excision repair [8]. With regard to its role, 5hmC is now recognized as a distinct epigenetic mark, closely connected to active genomic loci [9]. However, it remains to be elucidated whether 5fC and 5caC could act as true epigenetic marks by binding to their own reader proteins [10,11].

Most studies to date have focused on the DNA methylation landscape in deuterostomes (e.g., vertebrates) and ecdysozoans (e.g., arthropods, nematodes), and little information is available for lophotrochozoans, and especially mollusks—the most diverse phylum of this major clade of bilateral organisms [12]. DNA methylation occurs predominantly at cytosine-phosphate-guanine (CpG) sites and more rarely in non-CpG contexts, such as cytosine-phosphate-adenine (CpA), cytosine-phosphate-thymine (CpT), and cytosine-phosphate-cytosine (CpC) sites [13]. Many CpG dinucleotides are clustered together within regions of high CpG ratios and GC contents as “CpG islands” (CGIs) [14]. Such genomic areas are often located at the 5′-end of genes and contain DNA sequences to which relevant proteins (e.g., RNA polymerase, transcription factors) bind to begin transcription (promoters) and locations where the first DNA nucleotide is translated into RNA (transcription start sites) [15,16]. The number and density of CGIs vary widely across mammalian genomes, but it is estimated that 70–80% of CpG dyads in their somatic cells are generally methylated [17]. However, many constitutively expressed genes that are needed for maintaining basic cellular functions (housekeeping genes) lack methylation at their respective promoters [18]. The methylation status of these gene-regulatory elements is pivotal for modulating expression in genes responsive to biotic and abiotic stressors (inducible genes) [19]. Unlike mammals, the most common pattern encountered in invertebrates is mosaic methylation, with regions of heavily methylated DNA interspersed with non-methylated regions [20,21]; and gene-body methylation (GBM)—the ancestral DNA methylation pattern with methylation marks found primarily in the coding regions (exons) and non-coding regions (introns) of the gene transcriptional part (gene body) [11]. GBM exerts key roles in invertebrate genome functioning, such as transcription control, suppression of intragenic promoter activity, and inclusion/exclusion of exons into (from) pre-mRNa molecules (alternative exon splicing) [22,23]. It is also known that inducible genes across different invertebrate taxa tend to be less methylated than other gene categories (e.g., housekeeping genes) [24].

Methylation of cytosine to 5mC is the most abundant cytosine modification variant and about 95% exists in a CpG sequence context. Genome-wide 5mC content ranges widely across different animal clades [25], with invertebrates generally exhibiting intermediate methylation levels of around 2–15% [22]. As a general rule, 5mC is found in repetitive sequences and the gene body, but it is absent at CGIs within housekeeping promoters and other gene regulatory units [24]. This evolutionary conserved epigenetic mark is of paramount importance for spatiotemporal modulation of gene expression, decreasing the “noise” of unspecific transcription, and genome stabilization [26,27]. The first derivative of 5mC, 5hmC, is another evolutionary preserved epigenetic mark found across diverse animal groups [28]. Similarly, this influential node for epigenetic communication is present at much lower levels around the transcription start sites within gene promoters [29]. Rather, 5hmC is distributed within the body of genes and is thought to be mainly related to the fine-tuning of gene expression during embryogenesis and in neuronal cells [29,30]. The further oxidation products of 5hmC, 5fC, and 5caC occur in minute amounts in mammalian cells; that is, 10–100-fold less than the 5hmC percentage [31]. Experimental evidence suggests that, besides serving as indicators of final stages of 5mC demethylation, these cytosine methylation variants can affect transcription via enhanced pausing, backtracking, and decreased fidelity of RNA polymerase II [32].

The phylum *Mollusca* typically includes seven living classes, viz. *Polyplacophora* (chitons), *Aplacophora*, *Monoplacophora*, *Bivalvia* (oysters, mussels, clams, and scallops), *Cephalopoda* (squid, octopus, nautilus), *Scaphopoda* (tusk shells), and *Gastropoda* (snails, slugs, limpets). Besides their economic and medical importance, the animals in class *Gastropoda* constitute over three-quarters of the total number of mollusk species and are key players in ecosystems as primary consumers, predators, recyclers, disease carriers, parasite hosts/intermediaries, and pollinators [33]. From a scientific perspective, these invertebrates closely adhere to the preconditions of serving as suitable biological indicators due to their ability to accumulate and tolerate substantive concentrations of toxic environmental contaminants in their soft tissues without showing any major metabolic disorders [34,35]. Gastropods are also valuable study systems for neurobiology, immunology, and phylogenetics [33,36,37,38]. From a practical point of view, these mollusks have well-known physiology, are easily reared under controlled laboratory conditions, and are available as inbred lines. Moreover, many species are hermaphrodites, and as a consequence, their use is a more cost-effective alternative to gonochoric animal models (species with at least two separate sexes), such as mammals or earthworms [35].

Current literature data suggest that we have barely scratched the surface of DNA methylation in gastropods and other mollusks [23]. For example, Fallet et al. (2020) have reviewed data on different epigenetic marks and mollusks and found that this biologic process has been directly investigated in only 16 species belonging to three out of the seven molluskan classes: eight gastropods, seven bivalves, and one cephalopod [4]. In the meantime, however, this topic has attracted increasing research interest, fueled especially by the increasing availability of large sequence data sets and high-throughput methods for DNA methylation analysis, as reviewed by Fallet et al. (2023) [23]. In this review, we first focus on what is known to date concerning the components of the DNA methylation machinery in snails. Next, we examine the variability of DNA methylation in different species and tissues and discuss the potential roles of methylome dynamics in gastropod reproduction, growth, development, learning, memory, and immunity. We also summarize and examine studies connecting DNA methylation and environmental pollutants via gastropod models and discuss their implications for environmental risk assessment. In conclusion, the state-of-the-art and the related future challenges are provided. The present review hence aims to expand our knowledge and develop scientific interest in DNA methylation in this important, but largely understudied molluskan clade—a missing piece of a larger puzzle that shows how invertebrates may interact with their environment.

## 2. Components of DNA Methylation Machinery

### 2.1. Writers of DNA Methylation

DNA methyltransferases (DNMTs) are key enzymes in the process of DNA methylation, as they catalyze the transfer of a methyl group to DNA by flipping the target base out of the double helix [39]. In mammals, there are three major DNMT classes: DNMT1, DNMT2, and DNMT3A/3B/3L. DNMT1 is found in stem cells and adult cells and ensures the continuity of DNA methylation patterns by binding to hemi-methylated CpG sites and methylating the cytosine on the newly synthesized strand [40]. The DNMT3 family members (primarily DNMT3A and DNMT3B) initiate DNA methylation by adding methyl groups to previously unmethylated CpG sites, being pivotal for establishing de novo DNA methylation patterns during development and reproduction, as well as in response to environmental signals [39]. DNMT2 is the most widely conserved DNMT across animal phylogeny and contains all conserved motifs of cytosine DNA methyltransferases. However, it appears to be associated with DNA methylation in non-CpG contexts or specific genomic regions and primarily acts as an RNA methyltransferase by methylating only transfer RNA (tRNA) [40].

Indirect proof of the existence of DNMTs in gastropods was obtained from the sea hare *Aplysia californica* (Cooper, 1863), the marsh snail *Biomphalaria glabrata* (Say 1818), the bladder snail *Physa acuta* (Draparnaud, 1805), the Turkish snail *Helix lucorum* (Linnaeus, 1758), and the great pond snail *Lymnaea stagnalis* (Linnaeus, 1758) via addition of various demethylating agents, such as N-Phthalyl-L-tryptophan (RG108), 5-azacytidine (5-aza-C), 5-aza-2′-deoxycytidine (5-aza-dC), and 4-Deoxyuridine (zebularine) [38,41,42,43,44,45,46,47,48,49,50,51,52,53]. Direct evidence for the presence of DNMTs and their encoding genes derives from the analysis of genomic, proteomic, and transcriptomic datasets from *Biomphalaria glabrata* [38,54,55], *Lottia gigantea* (G.I. Sowerby I, 1834) [56,57], *Aplysia californica* [56,58,59,60], *Tritia mutabilis* (Linnaeus, 1758) [61], and *Cornu aspersum* (Müller, 1774) [62].

After conducting a search against the preliminary genome assembly v4.3 of *B. glabrata* [55], Fneich et al. (2013) identified the presence of protein-encoding sequences highly similar to those of the human *DNMT1*, as well as partial transcripts corresponding to the candidate gene [54]. Geyer et al. (2016) later described the structure of this gene [38]. Genes sharing a common ancestry (homologs) with the human *DNMT1* and its methyltransferase associating protein (DMAP1) were detected in the expressed sequence tags database of the neuronal transcriptome of *A. californica*, but there was no indication for the presence of *DNMT3* [58]. By using RT-PCR and primers specific to the human *DNMT* genes, Draghici et al. (2021) found pertinent evidence for the presence of *DNMT1* gene, but not of the *DNMT3* gene, in the genome of the brown garden snail *Cornu aspersum* (Müller, 1774) [62]. The former gene was also identified in the mutable nasa *Tritia mutabilis* (Linnaeus, 1758) [61]. The analysis of the compact genome of the owl limpet *Lottia gigantea* (Sowerby, 1834) revealed, by contrast, the occurrence of both *DNMT1* and *DNMT3* genes [56]. These data show that gastropods tend to present *DNMT1* homologues and lack *DNMT3* homologues in their genomes. Indeed, Männer et al. (2021) confirmed this trend after analyzing data from published genomes and transcriptomes of 140 species across all molluskan classes, including 23 gastropod species. More precisely, they examined the following species: *Aplysia californica*, *Melibe leonina* (Gould, 1852), *Phylliroe bucephala* (Lamarck, 1816), *Clione limacina* (Phipps, 1774), *Limacina antarctica* (Woodward, 1854), *Limacina helicina* (Phipps, 1774), *Limacina retroversa* (Fleming, 1823), *Siphonaria pectinata* (Linnaeus, 1758), *Elysia chlorotica* (Gould, 1870), *Elysia cornigera* (Nuttall, 1989), *Elysia timida* (Risso, 1818), *Radix auricularia* (Linnaeus, 1758), *Plakobranchus ocellatus* (van Hasselt, 1824), *Biomphalaria glabrata* (Say, 1818), *Biomphalaria pfeifferi* (Krauss, 1848), *Lymnaea stagnalis*, *Physella acuta*, *Arion vulgaris* (Moquin-Tandon, 1855), *Achatina fulica* (Férussac, 1822), *Bradybaena similaris* (Férussac, 1821), *Littorina saxatilis* (Olivi, 1792), and *Cepaea nemoralis* (Linnaeus, 1758) [21]. Given the critical role of DNMT1 in maintaining DNA methylation patterns [40], these findings lend support for a high degree of conservation of CG maintenance methylation machinery across different gastropod taxa.

Growing evidence suggests that *DNMT1* can exhibit tissue-specific and dynamic expression in snails and slugs. For *B. glabrata*, transcript levels in the ovotestis were significantly above those observed in the buccal mass, stomach, hepatopancreas (*syn.* digestive gland), salivary glands, albumen gland, kidney, central nervous system, heart, head/foot, and mantle edge [38]. Hemocytes, which are the invertebrate equivalent of mammalian phagocytes [36], also revealed consistently increased transcript abundance for this gene when compared to the albumen gland, stomach, head/foot, ovotestis, and hepatopancreas [38]. In addition, changes in *DNMT1* expression varied widely during the early stages of *T. mutabilis* embryo development [61]. These results point to DNA methylation maintenance as an important player for gastropod reproduction, development, and immunity. Given the pivotal role of *DNMT3* in development (as described above), it is likely that the apparent low frequency of *DNMT3* in gastropods does not necessarily imply that de novo methylation is rare in these invertebrates. It was hypothesized that *DNMT1* may regulate both the maintenance of methylation and de novo methylation, counterbalancing the lack of *DNMT3* [26,63]. This gene might therefore be finely tuned to respond to different cellular and environmental cues in various tissues, thus explaining its dynamic, tissue-specific expression in *B. glabrata* [36].

### 2.2. Readers of DNA Methylation

The readers of DNA methylation include three families of proteins that allow the translation of DNA modifications into functional transcriptional signals that regulate gene expression: methyl-CpG-binding domain (MBD) proteins, zinc-finger domain proteins, and ubiquitin-like proteins, containing PHD and RING finger domains (UHRF) [64]. MBD proteins are closely linked to transcriptional repression by recruiting chromatin remodelers, histone deacetylases, and methylases associated with this function. These proteins are able to bind to DNA containing at least one symmetrically methylated CpGs, but possess only a negligible non-specific affinity for unmethylated DNA [65]. The most important and well-characterized members of this family in mammals are MECP2, MBD1, MBD2, MBD3, and MBD4. MECP2, MBD1, and MBD4 regulate transcriptional repression, whereas MBD2 and MBD3 can function both by activating and reducing transcription levels. The majority of these enzymes have a high binding affinity for 5mC, except for MBD3, which has a similar binding preference for both 5mC and 5hmC [65,66]. The zinc-finger family of proteins comprises ZBTB4, ZBTB38, and Kaiso. The former two members can bind to a single methylated CpG and are highly expressed in the brain. Kaiso, by contrast, preferentially binds to two successively methylated CpG dinucleotides. Similar to MBD proteins, their role is associated primarily with inhibiting transcription in a DNA-methylation-dependent manner [67]. Members of the UHRF family are multidomain proteins, including UHRF1 and UHRF2 as the most well-studied members of the group. UHRF1 specifically recognizes and binds hemimethylated DNA and recruits DNMT1 to maintain DNA methylation patterns. In contrast, UHRF2 does not facilitate DNA methylation maintenance, despite sharing structural homology with UHRF1 [68].

Most information related to gastropods and readers of DNA methylation is available for MBD proteins. Moroz et al. (2013) was the first to provide relevant evidence for their existence; they identified a protein sharing 98% identity with the binding domain of the human MBD2 in the central nervous system of *A. californica* [60]. In *B. glabrata*, bioinformatic analysis revealed the existence of homologues for the *MBD2* and *MBD3* genes, but not for the *MBD1* and *MBD4* genes [45]. It was later confirmed that, like other invertebrates, this planorbid differs from vertebrates by having a gene fusing the functions of *MBD2* and *MBD3* genes (*MBD2/3* gene), but no separate genes. This gene displayed tissue-specific expression patterns, with transcript levels being consistently enriched in gonadal tissues (ovotestis, terminal genitalia) and hemocytes relative to other tissues, such as the buccal mass, stomach, hepatopancreas (*syn.* digestive gland), salivary glands, albumen gland, kidney, central nervous system, heart, head/foot, and mantle edge [38]. As a result, this gene may be involved in epigenetic regulation of gastropod reproduction and immunity.

### 2.3. Erasers of DNA Methylation

The ten-eleven translocation enzymes include TET1, TET2, and TET3. Despite harboring the same catalytic activity related to the conversion of 5mc into 5hmC via oxidizing the methyl group of 5mC [69], these proteins have distinct roles in mammals. Expressed during early development, TET1 and TET2 regulate transcriptional activation/repression and tumor suppression, respectively. In contrast, TET3 is mainly expressed in oocytes and fertilized zygotes, being involved in DNA methylation reprogramming [70]. Genomic sequences matching 50% of oxygenase domains specific to TET enzymes were found in *B. glabrata*, but no transcripts similar to the TET human orthologs (genes related via speciation) [54]. Therefore, it remains to be precisely assessed which members of *TET* family of enzymes or proteins with similar oxygenase activity do exist in gastropods and what their precise role is.

## 3. DNA (Hydroxy)methylation Levels in Gastropods

A comprehensive overview of up-to-date information on global, genome-wide DNA (hydroxy)methylation levels across different snail species and tissues are given in Table 1. These data show that gastropods tend to exhibit low to moderate cytosine methylation. At the whole-body level and for the organ/tissues examined, the percentage of 5mC displays relatively little inter-species variation, ranging between 0.1 and 4.9%—as measured via ELISA—and lies at the lower end of the range reported for invertebrate study systems—as reviewed by Šrut (2021) [22]. Limited information available on other cytosine modification variants support the presence of minute amounts of 5hmC in the gastropod genome (see Table 2). This methylation form was first identified in *P. glabrata*, with the measured levels being very low (see Table 2). Similar values were reported in *Aplysia* neurons [60], and possibly in *C. aspersum* hepatopancreas [71].

## 4. Role of DNA Methylation Machinery in Gastropods

As an essential player in the regulatory landscape of genetics, DNA methylation governs a myriad of biological processes, influencing everything from embryonic development to tissue homeostasis throughout an organism’s life span. The current knowledge of the role of DNA methylation machinery in mollusks derives mainly from bivalve studies, whereas other clades, including gastropods, played only a complementary role [81]. Analysis of *B. glabrata* methylome showed that cytosine methylation exists only in the CpG context [54]. Similarly, the Pacific abalone, *Haliotis discus hannai* (Inno, 1953), exhibited methylated cytosines mainly at CG dinucleotides (83%) and rarely at CHH and CHG trinucleotides (where H is thymine, adenine, or cytosine) [82]. Both *B. glabrata* and *L. gigantea* revealed global DNA methylation patterns consisting of regions of strong methylation alternating with regions of ultra-low methylation, with housekeeping genes being hypermethylated and inducible genes being poorly methylated [20,54,83]. As a general rule, snails possess substantial DNA methylation at the gene body [21]. The regulation of gene expression in these mollusks is mainly related to GBM [54,75,80,84], whereas indications regarding the regulatory effects of different promoter methylation status is rather unclear [4]. These data indicate that the DNA methylation landscape in these mollusks is consistent with that observed in other invertebrates; more precisely, it is of a “mosaic” type and occurs preferentially in a CpG context, primarily within the transcriptional units of genes with housekeeping function.

### 4.1. DNA Methylation Is an Important Player in Reproduction, Growth, and Development

From the earliest stages of germ-cell development to the intricate choreography of fertilization and beyond, DNA methylation serves as a sentinel, modulating gene expression, imprinting patterns, and ensuring the fidelity of genetic information transfer [1]. Its dynamic presence in reproductive processes is crucial, shaping the landscape of fertility, embryonic development, and the transmission of genetic information across generations [14]. Geyer et al. (2016) found that exposure to the demethylating agent 5-aza-C significantly inhibited egg production and embryo development in the planorbid snail, *Biomphalaria glabrata* [38]. In a study with sexually mature specimens of *Cornu aspersum*, Drăghici et al. (2023) reported significant hypermethylation of the ovotestis compared to the hepatopancreas [79], possibly reflecting the more stringent regulation of genes crucial for reproductive processes in the former organ [83,85]. The foregoing authors also identified significantly elevated transcript levels for several key genes of the DNA methylation machinery in the gonadal structures of *B. glabrata* compared to somatic tissues, such as the head/foot, central nervous system, buccal mass, salivary glands, hepatopancreas, stomach, heart, kidneys, and mantle edge. More precisely, *DNMT1* was overexpressed in the ovotestis, *DNMT2* in the terminal genitalia, and *MBD2/3* in both organs [38]. From a reproductive point of view, the ovotestis and terminal genitalia display different, but complementary roles. The former organ is central to production and storage of gametes, both eggs (ova) and spermatozoa (sperm), within a single structure [86]. The latter organ provides the necessary structures and mechanisms for the transfer and utilization of sperm from partners to fertilize the eggs [86]. As *DNMT1* ensures the maintenance of DNA methylation during cell replication and inheritance of DNA methylation patterns [67], its overexpression in the ovotestis hints at its critical role for proper gametogenesis, genomic imprinting, and the overall stability of epigenetic information passed on to the next generation of gastropods. On the other hand, *DNMT2* appears to be related to DNA methylation in non-CpG contexts or specific genomic regions [83]. As a result, its overexpression in terminal genitalia may reflect targeted methylation changes in expressions of specific genes involved in sperm transfer, fertility, or structural development. Moreover, *MBD2/3* overexpression in ovotestis and terminal genitalia might be indicative of its involvement in the regulation of DNA methylation patterns of genes associated with sex determination, sex-specific traits, sexual development, differentiation, and reproductive functions in *B. glabrata*. There is indeed evidence that *MBD2/3* plays an important regulatory role in the reproduction and development of certain invertebrates, such as insects or nematodes [87,88,89].

Imposex involves the development of male reproductive organs in female gastropods (particularly those from the *Muricidae* family), indicating a disruption in normal reproductive processes [90]. Srut et al. (2023) reported that genome-wide DNA demethylation related to exposure to tributyltin (TBT) is associated with a higher incidence of imposex in the banded murex, *Hexaplex trunculus* (Linnaeus, 1758) [91]. Although the DNA was extracted from foot tissues, these results support that cytosine methylation patterns may be particularly relevant in the context of reproductive genes and their dynamics may be linked to the aforementioned reproductive abnormalities.

Besides reproduction, dynamic changes of global, genome-wide DNA methylation accompany gastropod growth and development. Müller et al. (2016) reported that the whole-body DNA methylation in *P. acuta* decreased in a time-dependent manner after hatching (i.e., from 2.4% in 4-day-old juveniles to less than 0.15% in adult 200-day-old specimens) [78]. In contrast, global DNA methylation levels in the hepatopancreas of newly matured *C. aspersum* snails, aged 12 months, were relatively constant during a 56-day time frame [77]. The different life expectancies of these two gastropod species may account for these findings. That is, the long-lived *C. aspersum* (3–6 years) could display better mechanisms for DNA methylation maintenance compared to the shorter-lived *P. acuta* (7–12 months)—as already described in mammal species, e.g., bats, rodents [92,93].

Zhao et al. (2023) recently identified 383 age-associated methylated sites in specimens of the great pond snail, *Lymnaea stagnalis*, at four different life stages (1, 30, 90, and 200 days). DNA methylation levels at these sites varied with age and allowed discrimination between age classes [94]. The genetic material (DNA) analyzed in prior studies (see above) originated from tissues and cells (tissular DNA, herein abbreviated tDNA), whereas, for the latter study, environmental DNA (herein abbreviated eDNA) was examined. eDNA encompasses nuclear or mitochondrial DNA released by organisms into various environmental matrices, such as soil, water, air, or sediments, through processes like shedding skin cells, waste, excretions, shed hair, or decaying tissues [95]. Furthermore, Zhao et al. observed higher CpG methylation levels in the eDNA compared to the tDNA of *L. stagnalis* (10.73% versus 6.83%), with this distinction being age-specific and associated with a limited number of eDNA sites [94]. These findings suggest a potential mediation of age-related eDNA release via DNA methylation mechanisms.

Regarding gene-specific methylation patterns, evidence suggests that gene body methylation (GBM) plays a crucial role in modulating genes associated with snail growth and development. For example, Huang et al. (2021) studied the relationships between the transcriptome and methylome of *H. discus hannai*. Integrative analysis of 790 differentially expressed genes and 7635 differentially methylated genes revealed that GBM, but not promoter methylation, is essential to the regulation of genes related to growth (prolactin-signaling pathway, regulation of actin cytoskeleton) and development (neurotrophin signaling pathway) [82]. When combined with the aforementioned data, it can be inferred that overall DNA methylation patterns across the entire genome broadly influence the expression of genes involved in the reproduction, growth, and development of gastropod mollusks.

### 4.2. DNA Methylation as Essential Mediator of Learning and Memory

Besides the advantages of reduced time required for experiments and the low costs needed for their care, the gastropods’ biological and physiological attributes closely adhere to the preconditions of serving as suitable study systems for cellular and molecular neurobiological studies, particularly for studies of molecular mechanisms underlying learning and memory. First, these ubiquitous invertebrates have a more rudimentary brain structure compared to mammals, consisting of ganglia and clusters of neurons that perform basic functions, such as sensory processing, motor control, and simple forms of learning [96]. Second, their nervous system contains a smaller number of neurons (e.g., 10,000 to 20,000 in sea hares of *Aplysia* genus), with simpler neural circuits, with some of them being the largest in the animal kingdom [58]. Third, their range of reflex, motivated, and rhythmic behaviors can be modulated using basic forms of learning and memory [97]. For example, *Aplysia* displays simple reflexes and behaviors, such as gill and siphon withdrawal reflexes, which can be easily altered through conditioning experiments [60,98,99]. This simplicity in terms of size, complexity, neuronal diversity, specialized brain regions, sensory capabilities, and behavioral repertoire allows for easier experimental manipulations and enables scientists to observe the effects on memory and learning mechanisms more precisely.

Cellular mechanisms for neuronal integration, information encoding, and memory modulation in human and animal brains are based on evolutionary preserved DNA methylation/demethylation cycles [60,100]. Apart from insects [101], snails are the only invertebrates for which relevant data on this topic are available. Among gastropod species, one model system in which the role of DNA methylation in learning and memory has been extensively studied is the marine prosobranch *Aplysia californica* (Cooper, 1863). The presence of both 5mC and 5hmC in giant polyploid neurons of *A. californica* renders DNA methylation and demethylation as dynamic processes in the central nervous system (CNS) of these mollusks [60]. *Aplysia* neurons yield higher amounts of DNA compared to mammalian neurons, thus enabling researchers to efficiently profile the methylome of individual neurons [60]. Furthermore, this species exhibits long-term memory that can persist for weeks, making it an excellent model for investigating the molecular mechanisms underlying long-term memory (LTM) formation, storage, and retrieval [47,51,97,102]. Several studies with *A. californica* revealed that dynamic changes in neuronal DNA methylation modulate LTM. Thus, Rajasethupathy et al. (2012) identified a CpG island in the promoter of the memory-suppressor gene *CREB2* that commonly exists in both unmethylated and methylated states. This genomic region was almost completely methylated following serotonin exposure. The addition of the DNMT inhibitor RG108 caused its almost total demethylation, whereas methylation of this CpG island resulted in the enhancement of long-term synaptic facilitation [42]. Pearce et al. (2017) showed that administration of RG108 during (or shortly after) training to create a stable memory hindered LTM consolidation. Later inhibition, by contrast, eliminated the already consolidated LTM [47]. In addition, experiments with the same DNMT inhibitor rendered DNA methylation as critical for the consolidation of the memory for long-term sensitization (LTS) induced by noxious stimulation [97]. Moreover, inhibition of DNA methylation via RG108 or decitabine blocked sensitization and classical conditioning shortly (1 h) after the beginning of training [51].

Additional evidence for the role of DNA methylation in modulating LTM processes in gastropods derives from studies with members of the *Lymnaeidae* and *Helicidae* families. Laboratory experiments with an inbred laboratory strain (W-strain) of the great pond snail, *Lymnaea stagnalis*, showed that injection of 5-aza-C blocked enhanced LTM formation in mature snails exposed to memory-enhancing stressors, such as thermal stress (i.e., heat shock) or predators stress (i.e., crayfish effluent, shell clipping) [45,46,48,103]. The persistent effects (at least 4 weeks) of training juvenile snails from the same species in crayfish effluent also depended on DNA methylation [45]. In adult specimens of the Turkish snail, *Helix lucorum* (*Linnaeus*, 1758), injections of RG108 led to memory recovery at early stages of amnesia (3 h) when combined with conditioned food stimuli (reminder) induced via electrical stimulation every 15–20 min. [44,50]. Using adult specimens of *Helix lucorum taurica* (Krynicki, 1833), Zuzina et al. (2023) found that LTM is inhibited by the administration of the same non-nucleoside DNMT inhibitor, but was restored one day post-injection [53]. Overall, these data highlight the role of DNA methylation as a critical regulatory mechanism for memory-related processes within the snail’s CNS. Moreover, these findings emphasize the dynamic nature of these epigenetic modifications and their influence on the formation, consolidation, storage, and modulation of the snail’s long-term context memory.

### 4.3. DNA Methylation Is Responsive to Biotic Factors

Biotic stressors are living organisms or biological factors that exert stress on other living organisms. These include pathogens (viruses, bacteria, fungi, parasites), predators, competition among species, and symbiotic interactions. Biotic stressors can impact organism survival, growth, and reproduction by causing diseases, consuming tissues, or competing for resources [104]. Most studies on biotic stressors and DNA methylation in gastropod mollusks have focused on pathogens (especially parasites) and rarely on other biotic factors (e.g., predators). The interaction between the trematode parasite *Schistosoma mansoni* (Sambon, 1907) and *B. glabrata* is routinely used as a study system for understanding the host–parasite interplay, including how parasite stress impacts the snail’s DNA methylome. Ittiprasert et al. (2015) exposed specimens from the NMRI (New Mexico Resistant Intermediate) strain of *B. glabrata* to either thermal stress (pre-warmed water, 32 °C) or *S. mansoni* miracidia for 15 min, 30 min, 1 h, 2 h, and 5 h. Both exogenous stress factors caused cytosine DNA hypomethylation within the intragenic region of the locus encoding the heat shock protein 70 (HSP 70), but yielded distinct suppression patterns. Heat-treated specimens showed similar patterns of DNA methylation across different tissues (body tissues, hepatopancreas, head foot, and ovotestis), returning to normal values within 5 h post-exposure. Parasite-stressed snails, by contrast, exhibited a time-dependent decrease in hepatopancreas 5mC levels, whereas DNA methylation of the other tissues began to increase after this period of time [84]. The observed inter-organ differences in DNA methylation patterns due to *S. mansoni* suggest that different organs exhibit distinct roles or vulnerabilities in the host–parasite interplay. Indeed, the hepatopancreas is known to play an essential role for the development and multiplication of this parasite within its snail host [84].

Geyer et al. (2017) measured the expression of *DNMT1*, *DNMT2*, and *MBD2/3* in the hemocytes, albumin gland, head/foot, stomach, ovotestis, and hepatopancreas from mature specimens of the freshwater snail, *Biomphalaria glabrata*, infected with *S. mansoni* [38]. The measured values for *DNMT1* and *DNMT2* in hemocytes, which are key cells for the snail’s innate immune response to parasite infection [105], were much higher compared to those in other body parts [38]. *DNMT1* is central to preserving DNA methylation patterns during cell division [67], and hence, its elevated expression in these cells might imply a greater need for maintaining existing DNA methylation patterns in genes crucial for immune responses. This could involve perpetuating the methylation status of specific immune-related genes, influencing their expression during immune cell development, activation, and response to stimuli. Elevated *DNMT2* expression in hemocytes may also reflect its role in establishing new DNA methylation patterns or modifying existing ones in response to immune challenges [105]. This could involve targeting specific genomic regions associated with immune-related pathways or responses. Indeed, recent evidence indicates that in *H. discus hannai* GBM, but not promoter methylation, is essential for the regulation of the gene related to apoptosis [82].

In the aforementioned study, incubation of *B. glabrata* embryonic cell line with *S. mansoni* larval transformation products (herein abbreviated as LTP) resulted in significantly increased expression of two key genes for the DNA methylation machinery, i.e., the *DNMT1* and *MBD2/3* genes [38]. The upregulation of *DNMT1* provides pertinent evidence that *B. glabrata* is enhancing its DNA methylation maintenance machinery in response to *S. mansoni* infection. On the other hand, *MBD2/3* overexpression might modulate the mechanisms underlying the recognition of methylated DNA sequences as a part of the snail’s immune response. It may also be related to the presence of LTPs, which alter the aforementioned mechanisms as a method of manipulating the host’s epigenetic machinery. Taken together with data on expression of genes involved in the DNA methylation cycle in hemocytes versus other body parts, these genomic events support that the DNA methylation machinery is pivotal for the immune response of this snail species following *S. mansoni* infection. In addition, these findings underscore the dynamic response of this epigenetic machinery to parasitic challenges.

Joe (2003) investigated the effect of parasitic infection with trematodes *Philophthalmus* sp. or *Maritrema novaezealandensis* on genome-wide DNA methylation in Southern creeper, *Z. carinatus* (Sowerby II, 1855). No significant differences existed between different infection status groups. However, high variation existed among different infection status groups, such as 0.52% for uninfected gastropods, 0.58% for individuals infected with *M. novaezealandensis*, and 0.62% for specimens infected with *Philophthalmus* sp. [73]. These outcomes support that the effect of trematode infection on DNA methylation in snails may be species- and/or parasite-specific.

Although the interplay between gastropods and crayfish predators is relevant from ecological, behavioral, and physiological perspectives, little information exists on the methylomic effect of these interactions. It has been demonstrated that *L. stagnalis* detects and responds to the scent or the injury caused by a crayfish predator with multiple stress-related outcomes, including changes in neuronal DNA methylone (as described in Section 4.2). However, these data were obtained using indirect methods based on DNA methylation inhibitors, primarily 5-aza-C. Further studies are therefore required to expand on these findings and elucidate the epigenetic significance of these results.

Finally, DNA methylation in gastropod cells may be important for suppressing transcription of some transposable elements (*syn.* transposons or jumping genes; herein abbreviated as TEs). Given their ability to change their positions within a genome, TEs can serve as a source of genotoxic stress and affect cell identity and genome size by inducing mutations, altered gene expression, and chromosome rearrangements [106]. Whole-genome bisulfite sequencing (WGBS) analysis of the *B. glabrata* genome showed that many copies of the non-long terminal repeat (non-LTR) retrotransposon nimbus (BgI) are constitutively highly methylated [54]. This is the first transcriptionally active TE characterized from mollusks [107] and its activation (via demethylation) is thought to be associated with the infection and transmission of the parasitic blood fluke *S. mansoni* [54].

### 4.4. Alteration of DNA Methylation in Response to Abiotic Factors

Abiotic factors refer to non-living components of an ecosystem that affect living systems with respect to growth, maintenance, and reproduction. These factors include physical and chemical elements, such as temperature, sunlight, water, soil, pH levels, salinity, and atmospheric gases [104]. Their impact on DNA methylation in animals is diverse and complex, affecting various biological processes and phenotypic traits [52]. Current evidence supports that certain abiotic stressors can affect DNA methylation in gastropod mollusks. Thus, Kong et al. (2017) have investigated the combined effects of temperature and salinity on DNA methylation in juvenile specimens of Pacific abalone, *Haliotis discus hannai* Ino. A methylation-sensitive amplified polymorphism (MSAP) analysis was conducted on DNA samples extracted from the foot muscle of specimens reared at selected temperature (20, 24, and 30 °C) and salinity (22, 32, and 42 psu; i.e., practical salinity units). The treatments at 24 °C and 32 psu were taken into account as reference groups since these parameters corresponded to the best survival and growth rates. Although no significant changes in total 5mC content were observed, 67 and 63 genomic loci were found to be differentially methylated [108]. The WGBS analysis of two populations of the same gastropod species disclosed significant and targeted DNA hypomethylation in the gills of snails originating from the warmer area (Fujian province, South China) compared to individuals collected from the colder area (Liaoning province, North China) [82]. Bogan et al. (2020) exposed juvenile specimens of the thecosome pteropod *Limacina helicina antarctica* (Woodward, 1854) to seawater pCO_2_ of 255, 530, and 918 microatmospheres (μatm) for, respectively, 1, 3, and 6 days. Significant and reversible genome-wide DNA hypomethylation events, as assessed via ELISA, were detected in snails exposed to the greatest pCO_2_. In addition, the downregulated genes showed enrichment of body DNA methylation [80].

Understanding the intricate relationship between abiotic stress and DNA methylation is crucial in elucidating how organisms adapt to changing environmental conditions. Epigenetic variation is thought to mediate phenotypic plasticity and occur prior to genetic mutation accumulation [109]. Thorson et al. (2017) investigated the role of DNA methylation to adaptative variation in asexual populations of the New Zeeland mud snail *Potamopyrgus antipodarum* (Gray, 1843) collected from distinct habitats (two lakes and two rivers). The analysis of DNA from snail foot tissue through methylated DNA immunoprecipitation coupled with next-generation sequencing (MeDIP-seq) revealed habitat-specific numbers of differentially methylated regions (DMRs) and a significant association between the differences in shell shape phenotypic traits of *P. antipodarum* and the number of DMRs from different habitats [110].

More recently, the same authors examined differences in global DNA methylation in the foot of a single clonal lineage of *P. antipodarum* collected from lakes with different degrees of anthropogenic exposure (one rural lake and two urban lakes). Inter-habitat differences in the number of DMRs were identified, with these changes occurring both at intergenic and gene levels [111]. Moreover, elevated levels of 5mC, DNMT activity, and MBD-binding activity were detected in the head/foot of *B. glabrata* adults when comparing inbred with outbred (hybrid) populations [38]. These findings suggest that DNA methylation not only mediates phenotypic plasticity, but is also involved in maintaining heterosis (hybrid vigor) in gastropod mollusks.

### 4.5. DNA Methylation as an Emerging Tool in Ecotoxicology

Environmental contaminants are substances that are introduced into the environment and have harmful effects on living organisms, ecosystems, or the environment as a whole [104]. These contaminants, such as heavy metals, pesticides, and other toxic substances, can affect DNA methylation levels and patterns in both vertebrates and invertebrates [22]. From a toxicological point of view, DNA methylation provides more detailed information on local and global molecular events compared to traditional genetic endpoints (e.g., mutation accumulation, chromosomal aberrations) by revealing subtle and potentially reversible pre-transcriptional changes in gene regulation [112]. Because this epigenetic mark can be heritable and transgenerationally stable, it has the potential to reflect not only the effect of recent, but also those of the past and historic exposure events [22]. Modern methods for DNA methylation analysis enable precise, sensitive, fast, and accurate quantification of levels and patterns to which different forms of cytosine modification variants occur across different tissue and cell types [113]. Such advantages render the use of methylomic signatures as promising biomarkers in biomonitoring surveys and ecotoxicological hazard assessment.

In Table 2, we provide data on the effect of various environmental contaminants on DNA methylation in gastropods. We focus on results originating from controlled experiments, since this approach allows for precise control over environmental conditions, isolation of variables, and high replicability of obtained data [34]. Among different trace metals derived from anthropogenic sources, toxicological studies to date have focused on cadmium (Cd)—a potent modulator of the DNA methylome [79]. These experiments have used *C. aspersum* as a study system and were conducted under controlled laboratory environments using different cadmium salts as a source of Cd [77,78,79]. Cadmium induced an increase in the total 5mC content of hepatopancreas DNA, but only for relatively high exposure doses and times (as shown in Table 2). This effect seemed to be mediated by the type of inorganic anion bound to cadmium, with chloride ions having a stronger effect than either sulfate ions or nitrate ions [77,78,79]. The global DNA methylation levels in ovotestis, however, were not affected by exposure to cadmium nitrate [79] (as described in Table 2). These findings may be at least partly related to the role of hepatopancreas as the main organ of Cd storage and detoxification and could reflect organ- and cell-type-specific changes in methylation patterns after exposure to environmental contaminants [77].

**Table 2:** Effect of selected environmental contaminants on DNA methylation in gastropods.

Toxicant	Study System	Exposure Scenario (Duration, Route, Chemical Form, Dose, Specimen Age, Target Organ)	Methylomic Endpoint	Method	Main Results	Reference
Metals						
Cadmium (Cd)	*Cornu aspersum*	14-, 28-, and 56-day dietary exposure to 0, 0.05, 0.2, 1, 10 and 100 mg/L dietary Cd as CdCl_2_ (adult specimens, hepatopancreas)	Genomic 5mC content	Colorimetric ELISA	Significant and persistent hypermethylation starting at 28 days of exposure to 10 mg/L Cd	[77]
		28-day dietary exposure to 0, 0.05, 0.1, 0.2, 1, 10, and 100 mg/L Cd as CdSO_4_ (adults, hepatopancreas)	Genomic 5mC content	Colorimetric ELISA	Significant hypermethylation at 100 mg/L Cd	[78]
			Methylation status of 7 CpG sites at the 5′-UTR of the Cd-MT gene	MS-PCR	No effect	[78]
		28-day dietary exposure to 0, 0.05, 0.1, 0.2, 1, 5, 10, and 100 mg/L Cd as Cd(NO_3_)_2_ (adults, hepatopancreas)	Genomic 5mC content	Colorimetric ELISA	Significant hypermethylation at 100 mg/L Cd	[79]
		Idem above (adults, ovotestis)	Genomic 5mC content	Colorimetric ELISA	No effect	[79]
		28-day dietary exposure to 0, 0.05, 0.2, 1, 10 and 100 mg/L dietary Cd as CdCl_2_ (adult specimens, hepatopancreas)	Genomic 5hmC content	Colorimetric ELISA	Increase in 5hmC content at 1 mg/L Cd	[71]
Pesticide and endocrine disruptors						
Vinclozolin (VZ)	*Physa acuta*	Intergenerational effect: 24-h F0 cumulative exposure via water to 0, 0.01, 0.1, 10 and 100 µg/L VZ; effect measured in F1 (0, 15, 28 days; all doses) and F2 (0, 28 days; 0, 0.1, 100 µg/L VZ) for whole body	Genomic 5mC content	Colorimetric ELISA	No effect	[74]
		45-day cumulated exposure via water to 0, 0.0005, 0.005, 0.05, 0.5 and 5 mg/L VZ (adults, whole body)	Genomic DNA methylation	MSAP	Significant changes in DNA methylation patterns	[114]
Prednisolone (PDS)	*Physa acuta*	Multigenerational effect: F0–F2 cumulative exposure via water to 2, 4, 8, 16, 32, 64 µg/L PDS (adults, whole body)	Global DNA methylation	Colorimetric ELISA	Linear decrease of 5mC content in F1 generation	[76]

Several studies have also shown that environmental contaminants can affect DNA methylation in aquatic gastropods. It was thus found that vinclozolin affects genome-wide 5mC levels in the DNA of *P. acuta* in a dose- and time-dependent manner (Table 2). That is, a single acute exposure had no intra- or inter-generational effects [74], whereas longer exposure yielded significant changes in the DNA methylation patterns of parental (F0) specimens [114]. When exposed to prednisolone, this species revealed a dose-dependent decrease of 5mC content, but only in snails of the first filial generation (F1) [76]. As already mentioned above (see Section 4.1), TBT exposure was associated with reduced DNA methylation in the banded murex, *Hexaplex trunculus*, with global DNA demethylation being correlated with TBT tissue burden [22]. These data indicate that undirected and stochastic DNA methylation alterations related to such external insults could drive maladaptive biological outcomes in snails.

Cytosine methylation in gastropods appears to be less responsive to environmental contaminants compared to the accumulation of substances in tissues or organs over time. This was reported for the hepatopancreas of mature specimens of *C. aspersum* fed cadmium-enriched diets over a wide range of cadmium doses, exposure durations, and cadmium compounds [77,78,79]. Its sensitivity as a toxicological endpoint for cadmium exposure was, however, consistent with that observed for hypometabolism tendency and body weight changes [78,79]. Thus, global, genome-wide DNA methylation in gastropods may serve as a relevant biomarker for sublethal effects of exposure to certain environmental contaminants such as cadmium.

Little information also exists regarding the impact of environmental contaminants on cytosine methylation patterns/levels at specific loci or genes in the gastropod genome. Drăghici et al. (2023) investigated the effect of cadmium (given as cadmium nitrate) on the methylation status of CG pairs at the 5′ region close to the transcription site of the gene encoding the Cd-selective metallothionein (Cd-MT) in *C. aspersum*. A four-week dietary exposure yielded no changes in the methylation status of this locus for both the hepatopancreas and ovotestis (Table 2). However, studies on other invertebrates have shown that cadmium exposure can lead to gene-specific changes in DNA methylation patterns and levels. For example, Guan et al. (2019) found 39 demethylated genes and 24 hypermethylated genes in Cd-exposed specimens of *Drosophila melanogaster* compared to controls. These molecular events occurred at key genes for development, reproduction, and energy metabolism [115].

DNMT inhibitors (DNMTi) are routinely used to investigate the role of DNA methylation across different tissues and model systems. Among various demethylating agents, 5-aza-C, 5-aza-dC, RG108, and zebularine are the most common compounds used for this purpose [116]. As nucleoside analogues, these molecules must be first incorporated into the DNA/RNA to exert their effects. However, this integration is non-specific, leading to lower chemical stability compared to DNA nucleosides and causing toxic side effects even at relatively low concentrations [116]. For example, treatment with 5-aza-C significantly inhibited oviposition in *B. glabrata* [75]. Zebularine is more stable and has lower toxicity than both 5-aza-C and 5 aza-dC, but is not appropriate for DNA methylation modification in *B. glabrata*, despite being an effective inhibitor of DNA methylation in cancer cell lines and vertebrates [52,75,117]. After testing Flavodiiron protein 1 (Flv1) and Flavodiiron protein 2 (Flv2) as new non-nucleoside analogues DNMTi, Luviano et al. (2021) found that the former compound exerted transgenerational DNA methylation alterations in the aforementioned gastropod (see Table 2) while having no adverse effects on its survival and fecundity. The inhibitory efficiency of Flv1 was also confirmed for the physid snail, *P. acuta*, and the Pacific oyster, *C. gigas* [117]. These results favor the use of Flv1 in studies on DNA and/or multigenerational DNMTi experiments with gastropods and mollusks.

## 5. Conclusions and Future Perspectives for Research on Gastropod Methylome

The studies reviewed in this article indicate that gastropods possess a functional DNA methylation machinery. Since these mollusks tend to present *DNMT1* homologues, but lack *DNMT3* homologues, one can expect a high degree of conservation of CG maintenance methylation mechanisms across different gastropod taxa. Readers of DNA methylation include the *MBD2* and *MBD2/3* proteins. *DNMT1* and *MBD2/3* display tissue-specific, dynamic expression patterns, serving as key players in reproductive/development and immune processes. The gastropod genome contains low to moderate 5mC levels, with moderate inter-species and inter-organ variation. The TET-DNMTs interplay modulates the epigenetic landscape via reversion of DNA methylation by TET enzymes when gene expression is active [105]. Data related to DNA methylation erasers (i.e., *TET* genes) in snails are limited and inconclusive, despite the confirmed presence of small amounts 5hmC in the gastropod genome. If snails have very low levels of these enzymes (or enzymes with similar functions), as suggested by the available literature data [54], DNA methylation may be less reversible in these invertebrates, leading to a more stable DNA methylation pattern over time. This could result in the perpetuation of epigenetic marks across generations, potentially limiting the ability of gastropods to respond to environmental changes through epigenetic modifications. There is, indeed, evidence that DNA methylation reprogramming is a mammalian-specific feature not an invertebrate (non-mammalian) feature [118]. This supports the idea that invertebrates, including gastropods, possess a higher evolutionary potential within their epigenome compared to mammals.

The prevalence of CpG methylation, observed consistently across different gastropod species, indicates a common mechanism for genetic regulation in these organisms. The alternating patterns of methylation intensity, with housekeeping genes exhibiting higher methylation levels compared to inducible genes, hint at a nuanced regulatory mechanism finely tuned to maintain essential cellular functions while enabling flexibility for adaptation. The significant DNA methylation within gene bodies implies a crucial role for gene body methylation (GBM) in modulating gene expression, particularly for genes with fundamental roles in cellular processes. Overall, the mosaic-like DNA methylation landscape observed in gastropod mollusks mirrors the patterns seen in other invertebrates, suggesting evolutionary conservation of regulatory mechanisms across diverse taxa [24].

Acting as a critical regulator, from early germ cell development to fertilization, DNA methylation modulates gene expression, imprinting patterns, and ensures the fidelity of genetic information transfer. This epigenetic mark is central to gastropod reproduction, impacting egg production, embryo development, and sex-specific traits. Gonadal structures display tissue-specific patterns of DNA methylation and distinctive expression of key genes in the DNA methylation machinery (e.g., *DNMT1*, *DNMT2*, *MBD2/3*) compared to somatic tissues. Dynamic changes in global DNA methylation also accompany growth and development, with variations observed across species and life stages. Furthermore, gene-specific methylation patterns influence the expression of genes involved in growth, development, and reproduction. DNA methylation, hence, emerges as a central modulator, shaping the snail’s reproductive, growth, and developmental trajectories.

The interplay between DNA methylation and demethylation, both at gene-specific and genome-wide levels, serves as an important regulatory mechanism governing learning and memory formation, consolidation, and persistence in gastropods. DNA methylation is also responsive to biotic stressors, such as pathogens and predators. Differential DNA methylation patterns observed in various tissues of specimens exposed to parasite stress indicate organ-specific vulnerabilities or roles in the host–parasite interplay, with implications for immune responses and parasite development. In addition, these invertebrates exhibit changes in neuronal DNA methylation associated with stress-related responses to predator cues. However, the link between DNA methylation and transposable elements in gastropods exposed to biotic factors has not been investigated to date. Transposable elements are implicated in host-pathogen interactions by serving as targets for immune defense mechanisms and playing roles in the evolution of host resistance [119]. Investigating the relationship between DNA methylation, TEs, and biotic factors in gastropods can thus provide valuable insights into fundamental biological processes, evolutionary dynamics, and ecological interactions, with implications for both basic research and practical applications in conservation and management.

Abiotic factors, such as temperature, salinity, and seawater pCO_2_ levels, exert a significant impact on DNA methylation in gastropod mollusks. Exposure to different environmental conditions results in different DNA methylation patterns across their genome. DNA methylation is also involved in adaptive phenotypic variation (e.g., shell shape) and maintenance of heterosis. These findings highlight the intricate relationship between abiotic factors and DNA methylation in snails, emphasizing the importance of epigenetic mechanisms in mediating adaptation to changing environments and maintaining phenotypic diversity within populations. Various environmental contaminants, including metals (cadmium), pesticides (vinclozolin), and other toxic substances, can also influence DNA methylation levels and patterns in gastropods. These alterations are often dose- and time-dependent and tend to occur rather at global than at gene-specific level. In addition, these changes in DNA methylation patterns can precede or accompany other physiological responses to contaminant exposure. Global, genome-wide DNA methylation in gastropods may hence serve as a relevant biomarker for sublethal effects of exposure to environmental contaminants.

Unfortunately, there is a lack of comprehensive studies investigating how DNA methylation patterns change across different developmental stages, tissues, and environmental conditions in snails. Longitudinal studies tracking DNA methylation changes over time are hence needed to elucidate the temporal dynamics of epigenetic regulation in snails. A major drawback is the limited data available for gastropod DNA methylome. In fact, to the best of our knowledge, there is only one whole methylome available for these mollusks, i.e., for *B. glabrata* [120]. While studies have identified methylated regions in the snail genome, the functional significance of these regions is often unclear. Functional annotation of methylated regions, including their association with genes, regulatory elements, and phenotypic traits, is therefore necessary to understand the role of DNA methylation in snail biology and adaptation. There is also a need for integrated analyses combining genomic and epigenomic data to unravel the complex interactions between genetic and epigenetic factors shaping snail phenotypes. Integrative approaches should enable researchers to identify candidate genes, pathways, and regulatory networks underlying phenotypic variation and adaptation in snails.

## Figures and Tables

**Table 1:** Global DNA (hydroxy) methylation levels in gastropods.

Species	DNA Base	Percentage	Source	Method	Reference
*Helix pomatia*	5mC	2.9 *			[72]
*Patella* sp.	5mC	4.9 *			[72]
*Zeacumantus subcarinatus*	5mC	0.3–0.9	whole body	ELISA	[73]
*Aplysia californica*	5mC	1.5–3	abdominal ganglions	ELISA	[51]
*Physa acuta*	5mC	0.15–2.4	whole body	ELISA	[74]
	5mC	0.2–0.4	whole body	ELISA, Dot blot	[52,75]
	5mC	0.4–0.68	whole body	ELISA	[76]
*Cornu aspersum*	5mC	0.067–0.48	hepatopancreas	ELISA	[77]
	5mC	0.29–0.99	hepatopancreas	ELISA	[78]
	5mC	0.27–0.88	hepatopancreas	ELISA	[79]
	5mC	0.42–0.94	ovotestis	ELISA	[79]
	5hmC	<0.03	hepatopancreas	ELISA	[71]
*Biomphalaria glabrata*	5mC	2	foot	LC-MS/MS	[54]
	5mC	1.34–4.28	head, foot	ELISA	[38]
	5mC	1.74–1.94	whole body	Dot Blot	[75]
	5mC	0.5–2	whole body	ELISA	[75]
	5hmC	0.0009	foot	LC-MS/MS	[54]
*Limacina helicina antarctica*	5mC	0.5–1.3	whole body	ELISA	[80]

* Data reviewed from literature, but no details on the sampled tissue and the method used to quantify 5mC levels are provided.

## Data Availability

Not applicable.
